# Black Carbon Exposures, Blood Pressure, and Interactions with Single Nucleotide Polymorphisms in MicroRNA Processing Genes

**DOI:** 10.1289/ehp.0901440

**Published:** 2010-03-08

**Authors:** Elissa H. Wilker, Andrea Baccarelli, Helen Suh, Pantel Vokonas, Robert O. Wright, Joel Schwartz

**Affiliations:** 1 Department of Environmental Health, Harvard School of Public Health, Boston, Massachusetts, USA; 2 Cardiovascular Epidemiology Research Unit, Beth Israel Deaconess Medical Center, Boston, Massachusetts, USA; 3 Center of Molecular and Genetic Epidemiology, Department of Environmental and Occupational Health, Fondazione IRCCS Ca’ Granda Ospedale Maggiore Policlinico e Università degli Studi di Milano, Milan, Italy; 4 VA Normative Aging Study, Veterans Affairs Boston Healthcare System and the Department of Medicine, Boston University School of Medicine, Boston, Massachusetts, USA; 5 Channing Laboratory, Brigham and Women’s Hospital, Boston, Massachusetts, USA

**Keywords:** black carbon, blood pressure, epigenetic mechanisms, gene-environment interactions, genetic polymorphisms

## Abstract

**Background:**

Black carbon (BC) is a marker of traffic pollution that has been associated with blood pressure (BP), but findings have been inconsistent. MicroRNAs (miRNAs) are emerging as key regulators of gene expression, but whether polymorphisms in genes involved in processing of miRNAs to maturity influence susceptibility to BC has not been elucidated.

**Objectives:**

We investigated the association between BC and BP, as well as potential effect modification by single nucleotide polymorphisms (SNPs) in miRNA processing genes.

**Methods:**

Repeated measures analyses were performed using data from the VA Normative Aging Study. Complete covariate data were available for 789 participants with one to six study visits between 1995 and 2008. In models of systolic and diastolic BP, we examined SNP-by-BC interactions with 19 miRNA-related variants under recessive models of inheritance. Mixed-effects models were adjusted for potential confounders including clinical characteristics, lifestyle, and meteorologic factors.

**Results:**

A 1-SD increase in BC (0.415 μg/m^3^) was associated with 3.04 mmHg higher systolic (95% confidence interval (CI), 2.29–3.79) and 2.28 mmHg higher diastolic BP (95% CI, 1.88–2.67). Interactions modifying BC associations were observed with SNPs in the *DICER*, *GEMIN4*, and DiGeorge critical region-8 (*DGCR8*) genes, and in *GEMIN3* and *GEMIN4*, predicting diastolic and systolic BP, respectively.

**Conclusions:**

We observed evidence of effect modification of the association between BP and 7-day BC moving averages by SNPs associated with miRNA processing. Although the mechanisms underlying these associations are not well understood, they suggest a role for miRNA genesis and processing in influencing BC effects.

Exposure to particulate air pollution has been associated with cardiovascular morbidity and mortality in numerous epidemiologic studies (Brook 2008; Pope et al. 2004). Black carbon (BC), a combustion by-product, is a widely used marker of traffic pollution and has been linked to cardiac and ventricular arrhythmias (Rich et al. 2005), ST-segment depression (Gold et al. 2000), decreased flow-mediated vascular reactivity (O’Neill et al. 2005), lowered heart rate variability (Schwartz et al. 2005), and increased cardiovascular mortality (Maynard et al. 2007). Growing evidence suggests that traffic-related pollution, including BC, may be driving the cardiotoxic effects observed in response to air pollution exposures (Hoffmann et al. 2007). A few recent studies have examined associations between particles and blood pressure (BP), and although positive associations have been observed (Auchincloss et al. 2008; Ibald-Mulli et al. 2001; Zanobetti et al. 2004), both inverse (Harrabi et al. 2006) and null (Jansen et al. 2005; Madsen and Nafstad 2006) associations have also been reported.

MicroRNAs (miRNAs) are small, noncoding RNAs that repress or inhibit gene expression by targeting messenger RNA (mRNA) (Mattick and Makunin 2006; Zhang 2008). Evidence suggests that miRNAs affect pathogenic pathways including angiogenesis (Suarez and Sessa 2009), redox signaling (Brewer and Shah 2009; Urbich et al. 2008), and stress response (van Rooij et al. 2007). Much of the existing miRNA-related literature has focused on cancer outcomes (Esquela-Kerscher and Slack 2006; Hu et al. 2008, 2009; Luthra et al. 2008; Yang et al. 2008). However, there is growing evidence that the dysregulation of cell-signaling pathways associated with miRNA is a key factor affecting heart disease (Chen et al. 2008; Cheng et al. 2007; Divakaran and Mann 2008; Ikeda et al. 2007; Zhang 2008). Additionally, studies in controlled environments have demonstrated cardiovascular effects due to loss of function of miRNA processing genes (Asada et al. 2008; da Costa Martins et al. 2008; Suarez et al. 2007). For the current study, we genotyped participants for potentially functional single nucleotide polymorphisms (SNPs) involved in the processing and formation of miRNAs. We then investigated the association between air pollution and BP as well as potential differences in susceptibility by SNP carrier status. We hypothesized that polymorphisms in genes that regulate miRNA processing could modify effects of BC on BP, a marker of autonomic function and cardiovascular health.

## Materials and Methods

### Study population

Our study participants were members of the Veterans Affairs Normative Aging Study (NAS). This is an ongoing longitudinal study of aging established in 1963, details of which have been published previously (Bell et al. 1966). Briefly, the NAS began as a closed cohort of 2,280 male volunteers from the greater Boston area, 21–80 years of age at entry, who enrolled after an initial health screening determined that they were free of known chronic medical conditions. All participants provided informed consent. Collection of blood samples for genetic analysis began in the late 1990s; 942 participants were still actively participating and provided BP and blood samples for some or all miRNA-related SNPs, which were successfully genotyped. Participants were reevaluated every 3–5 years using detailed on-site physical examinations and questionnaires.

### Physical parameters and medical history

Study center visits occurred after an overnight fast and abstention from smoking. Physical examination included measurement of height and weight, with body mass index (BMI) calculated as weight (kilograms)/height (meters squared). Questionnaires evaluated lifestyle factors and medication use. Type 2 diabetes was classified based on physician’s diagnosis or fasting blood glucose > 126 mg/dL.

### BP measurements

At each clinical visit, a physician measured BP using a standard mercury sphygmomanometer with a 14-cm cuff. Systolic BP (SBP) and fifth-phase diastolic BP (DBP) were measured in each arm to the nearest 2 mmHg while the participant was seated. The means of the right and left arm measurements were used as the BP measurement of each participant for analytical purposes. Although there was no specific rest period prior to measurement of BP, SBP and DBP were measured immediately after a complete patient history was taken with the subject seated.

### SNP selection and genotyping

SNPs were selected based on previously published work investigating associations between genes involved in miRNA processing and disease (Horikawa et al. 2008; Yang et al. 2008). These SNPs were chosen because of overlap in pathways involved in cancer processes related to autonomic function through cell signaling, apoptosis, angiogenesis, and inflammation. Genotyping was performed using multiplex polymerase chain reaction assays designed with Sequenom SpectroDESIGNER software (Sequenom, Inc., San Diego, CA). The extension product was then spotted onto a 384-well spectroCHIP before analysis in the MALDI-TOF mass spectrometer (Sequenom, Inc.). Duplication was performed on 5% of the samples. The 24 SNPs analyzed for this study were all successfully genotyped. After genotyping, we excluded those SNPs for which fewer than 10 participants were homozygous variant carriers [rg1106042 in *HIWI*, rs3742330 in *DICER*, rs417309 in DiGeorge critical region-8 (*DGCR8*), rs636832 in Argonaute 1 (*AGO1*)] and also those with a Hardy–Weinberg *p*-value < 0.05 (rs10719 in *DROSHA*), leaving a total of 19 SNPs in 10 genes.

### BC and meteorologic measurements

Continuous BC was measured at a Harvard School of Public Health monitoring site located at Countway Library (10 Shattuck Street, Boston, MA, USA), 1 km from the clinical examination site, and was averaged by hour before BP measurement using an aethalometer (Magee Scientific, Berkeley, CA, USA). We obtained temperature and relative humidity measures from the Boston airport weather station.

### Statistical methods

Because repeated measures of BP were available for many of these study participants with both BC measurements and genotyping, we were able to obtain greater power by using multiple measures. We evaluated SBP and DBP as dependent variables and analyzed their associations with BC in linear mixed-effects models. Previous studies have suggested that longer averaging times are more relevant to the associations between particles and BP (Zanobetti et al. 2004), and recent sensitivity analysis within the NAS found 7-day moving averages as being most strongly associated with BP outcomes for short-term time windows (1 hr to 1 week) (Mordukhovich et al. 2009). Therefore, we used 7-day moving averages of ambient BC concentrations matched on the time of BP measurement for each participant, and we evaluated SBP and DBP as dependent variables.

We examined associations between BP and BC using two different approaches to address potential confounding. In model 1, we adjusted for age, BMI, BC, and apparent temperature (a marker of perceived temperature) as continuous variables as well as smoking status (never, current, former) and season of clinical visit (spring: March–May; summer: June–August; fall: September–November; winter: December–February). In model 2, we adjusted for covariates included in model 1 as well as blood urea nitrate, pulse, and median income treated as continuous variables in addition to education (≤ 12 years, 13–16 years, and > 16 years), alpha blocker, beta blocker, calcium channel blocker, angiotensin receptor blocker, angiotensin-converting enzyme (ACE) inhibitor, diuretic use, two or more alcoholic drinks per day, and diabetes diagnosis. We chose to examine recessive models of inheritance only (rather than dominant and additive) to limit the number of associations tested. Covariates from model 2 were selected *a priori* to be included in the regression models, regardless of statistical significance. We then examined the associations between the 19 SNPs in miRNA processing genes as well as SNP-by-BC cross-product terms to assess interactions. The default α level was defined as *p* = 0.05.

We performed sensitivity analyses to determine whether our results were robust to changes in the covariates adjusted for in the models and also whether there were significant effects due to missing data. To address attrition in the study, we used inverse probability weighting to determine whether reweighting these observations with only one or two repeated measures significantly changed our results (Hernan et al. 2006). This was done by modeling the probability of having only one measurement or only two measurements using the covariates described in model 2 and treating those participants with three or more visits as the reference group. We then used the inverse of the predicted values as the weights. We also examined associations adjusting only for covariates selected in model 1 to determine whether the significance and or magnitude of our adjusted results changed. Linkage disequilibrium (LD) between SNPs in the same gene that met the significance criterion were assessed using the LDPlotter tool (Innate Immunity in Heart Lung and Blood Disease; http://www.pharmgat.org/IIPGA2/Bioinformatics/).

## Results

Of the 2,280 men who originally entered the cohort in 1963, complete covariate data were available on participants who took part in one to six examinations during the study period. Because the NAS comprises over 95% white participants, we restricted our analysis to white individuals based on self-report. There were 942 participants with some or all microRNA-processing genotyping data and BP measurements. Of these, BC data were available for 799 participants who reside in Massachusetts. All visits occurred between 1995, when pollutant monitoring began, and 2008. Our full models (model 2) include data from the 789 participants with complete covariate data and some or all genotyping data. In this group, 645 (82%) participants had at least two study visits from 1995 to 2008; 475 (60%) had three or more visits. Our study population was composed entirely of males, most of whom were former cigarette smokers (Table 1). The mean age (± SD) of study participants was 72.3 ± 7.5 years and mean BMI was 28.0 ± 4.1 kg/m^2^. Average SBP and DBP were 132 ± 18.4 mmHg and 76.8 ± 10.9 mmHg, respectively. We evaluated the association of SBP and DBP with ambient BC and expressed the results as the mmHg change associated with a 1-SD increase in BC (equivalent to 0.415 μg/m^3^) (Table 2). In our fully adjusted models (model 2), we found that a 1-SD increase in BC concentration was associated with 3.04-mmHg higher SBP (95% CI, 2.29–3.79; *p* = 0.003) and a 2.28-mmHg higher DBP (95% CI, 1.88–2.67; *p* < 0.001). These associations were attenuated compared with our model 1 analyses, which were adjusted for a subset of potential confounders. In model 1 analyses, we observed that a 1-SD change in BC was associated with 3.52-mmHg (95% CI, 2.77–4.26) and 2.72-mmHg (95% CI, 2.31–3.12) changes in SBP and DBP, respectively.

The complete list of the 19 SNPs analyzed is described in Table 3. We genotyped 24 SNPs and excluded those SNPs that, in fewer than 10 study participants, were homozygous carriers of the variant allele (rg1106042 in *HIWI*, rs3742330 in *DICER*, rs417309 in *DGCR8*, rs636832 in *AGO1*) and those in which Hardy–Weinberg equilibrium was not met at the 0.05 level (rs10719 in *DROSHA*), leaving a total of 19 SNPs in 10 genes. First, we examined the main effects of the SNPs of interest within this population. In our fully adjusted models, none of these SNPs were associated with BP at the 0.05 level. We examined BC-by-SNP interactions, and results are reported in Table 4. In models of SBP, interactions with BC were observed for two SNPs. For homozygous variant carriers of rs197414, a 1-SD change in BC was associated with 9.82-mmHg lower DBP (95% CI, −18.68 to −0.95), whereas in wild-type individuals and heterozygous carriers, we observed a 3.07-mmHg increase in BP (95% CI, 2.32–3.82); however, there were only 10 homozygous carriers of this variant, and the CI of these carriers is very wide. We also observed 5.58-mmHg higher BP (95% CI, 3.01–8.14) among *GEMIN4* rs1062923 homozygous recessive carriers in response to a 1-SD change in BC, but only 2.87 (95% CI, 2.10–3.65) in heterozygotes and homozygous wild-type carriers. Because this SNP had a Hardy–Weinberg *p*-value of 0.05, we approach interpretation of these results with caution.

We observed statistically significant interactions for four SNP-by-BC interactions predicting DBP. In all of these analyses, smaller changes in BP were observed for carriers of the homozygous variant genotype. A 1-SD change in BC was associated with 1.48-mmHg higher DBP (95% CI, 0.78–2.18) for homozygous variant carriers and 2.57 mmHg (95% CI, 2.12– 3.02) in others. The rs13078 *DICER* SNP was associated with 0.13-mmHg higher BP in carriers of the homozygous variant (95% CI, −1.65 to 1.91) and 2.32 (95% CI, 1.91–2.72) in others. Under a recessive model, homozygous carriers of the variant alleles of rs7813 and rs910925, both in *GEMIN4*, were both associated with lower BP in response to a 1-SD increase in BC compared with others. Both these SNPs produced very similar magnitudes of effect and were found to be in high LD (*r*^2^ = 0.9). As a sensitivity analysis, we also investigated whether there were significant associations in models adjusted only for model 1 variables, and the same SNPs achieved significant results. We also tested our associations using inverse probability weighting (IPW) to address attrition and found that our most significant results for both SBP and DBP became more significant, although changes associated with the IPW analysis were modest. We report the model 1 *p*-values and IPW *p*-values for sensitivity additionally in Table 4.

## Discussion

We observed that BC concentration averaged over the 7 days preceding each study center visit was positively associated with SBP and DBP in a cohort of elderly men. Furthermore, SNPs in miRNA processing genes modified the BC associations with BP that we observed. Our analysis focused on potentially functional SNPs located in genes involved in the processing of miRNAs. To our knowledge, this is the first study to investigate the role of polymorphisms in miRNA processing genes to be identified as effect modifiers of an association between air pollution and cardiovascular response.

**Figure 1:**
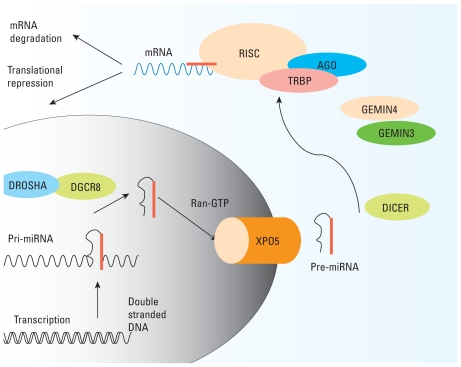
Major steps in miRNA processing. Long primary transcripts coded within the introns of protein-coding genes are transcribed into primary miRNAs (pri-miRNAs). Then DROSHA and the DGCR8 complex mediate the formation of the pre-miRNA stem-loop. After export from the nucleus via the *RAN-GTP* complex and Exportin*5*, these pre-miRNAs undergo modification by *DICER* within the cytoplasm to generate an ~ 22-nt duplex from the loop complex, which comprises the miRNA and its complement. AGO2, GEMIN3, and GEMIN4 then interact with miRNA to form a ribonucleoprotein that guides the miRNA into the RISC, where the miRNA strand anneals to the 3′ UTRs of target mRNAs, promoting translational repression or mRNA degradation.

We selected these SNPs because of their involvement in the biogenesis and processing of miRNA. The transcription and processing of miRNAs has been described in detail elsewhere (Bartel 2004). Briefly, miRNAs are processed in a multistep pathway (Figure 1). First, long primary transcripts coded within the introns of protein-coding genes are transcribed into primary miRNAs (pri-miRNAs) approximately 100 nucleotides (nt) in length. Through a series of steps mediated by DROSHA and DGCR8, the pre-miRNA stem-loop (approximately 70–100 nt) is formed. After export from the nucleus via the RAN-GTP complex and XPO5, these pre-miRNAs undergo modification by DICER within the cytoplasm to generate an ~ 22-nt duplex from the loop complex, which comprises miRNA and its complement. AGO2, GEMIN3, and GEMIN4 then interact with miRNA to form a ribonucleoprotein, which guides the miRNA into the RNA-induced silencing complex (RISC), where the miRNA strand anneals to the 3′ untranslated regions (UTRs) of target mRNAs, promoting translational repression or mRNA degradation. More than 700 human miRNA have been identified in the miRBase Registry (http://microrna.sanger.ac.uk/) (Griffiths-Jones et al. 2008).

It has been shown that miRNAs, which are generally associated with silencing or suppression of target gene transcripts (negative regulation), may become activators of gene expression during stress (Rocha 2007; Yang and Paschen 2008). Animal studies have also shown that miRNA expression is downregulated in lungs of rats exposed to cigarette smoke, which is consistent with the hypothesis that alterations of the miRNA control mechanism can occur even after relatively short periods of exposure (Izzotti et al. 2009). Other forms of epigenetic regulation, such as DNA methylation, have been associated with exposure to traffic particles (Baccarelli et al. 2009). Recently, miRNA processing genes, such as *DICER*, have been associated with endothelial function and angiogenesis (Suarez et al. 2007).

None of the interactions identified were significantly predictive of both SBP and DBP. Although BC interactions with SNPs in *DGCR8*, *DICER*, and *GEMIN4* were observed in models of DBP, only interactions with single SNPs in *GEMIN3* and *GEMIN4* were predictive of SBP. The *DICER* and *DGCR8* SNPs for which we observed interactions predicting DBP are both located within 3′ UTRs. These regions are not translated directly into the protein; however, they are thought to play a role in mRNA stability. Additionally, these polymorphisms may affect miRNA binding to the 3′ UTR of target mRNAs. Previously, *DICER* knockdown increased activation of the endothelial nitric oxide synthase pathway (Suarez et al. 2007). DICER is thought to be more generally involved in miRNA processing, whereas DGCR8 is thought to be more specific to particular miRNAs (Han et al. 2006). *DGCR8* is required for the maturation of miRNA primary transcripts at the initial stages. A recent study found that deletion of *DGCR8* in cardiomyocytes leads to left ventricular malfunction progressing to a dilated cardiomyopathy and premature lethality (Rao et al. 2009), which suggests a central role of this gene in cardiovascular function. Lack of DICER activity in knockout studies has been suggested to be associated with defective blood vessel formation (Bernstein et al. 2003; Yang et al. 2005). However, no associations predicting SBP have been observed for the particular SNPs that we observed interacting with BC.

In our analyses, we found that BC interactions with SNPs in *GEMIN4* were associated with SBP and DBP. Interactions with one SNP in *GEMIN3*, rs197414, was also associated with SBP. These genes code for proteins associated with the survival motor neuron (SMN) complex. GEMIN3 is a DEAD-box RNA helicase that binds directly with the SMN, and GEMIN4 is thought to play a role as cofactor in this response (Charroux et al. 2000). Together, GEMIN3 and GEMIN4 play a critical role in the processing of pre-miRNA by introducing the miRNA precursor to the RISC (Murashov et al. 2007). Along with the AGO proteins, these genes form a complex with the miRNA to create a ribonucleoprotein (Maniataki and Mourelatos 2005). Previously, the *GEMIN3* SNP rs197414, for which we observed much lower BP among the few carriers, has been associated with higher bladder cancer rates (Yang et al. 2008). A study found that the *GEMIN4* rs7813 variant was associated with *in vitro* hepatocellular carcinoma cell line growth inhibition compared with the wild-type allele, suggesting that the amino acid change caused by this SNP might have physiologic significance (Wan et al. 2004).

Some mechanisms of BC effects may be different for SBP and DBP. For example, other studies have observed stronger associations between ambient pollutant exposures and DBP as opposed to SBP in human (Fakhri et al. 2009) and animal studies (Bartoli et al. 2009) in controlled environments. The mechanisms for these differences are not well understood. Systolic pressure rises with age; by contrast, diastolic pressure increases until around age 50 years and falls with increasing years (Burt et al. 1995). The role of SBP and DBP in predicting downstream health consequences remains controversial. Some recent reports have suggested that SBP should be the primary risk factor reported in older individuals (Williams et al. 2008). However, within the Framingham Heart Study, isolated diastolic hypertension was a cardiovascular risk factor (Franklin et al. 2009). Subjects with this condition were found to have about two times the cardiovascular risk with normal BP, and increased cardiovascular risk among subjects with isolated diastolic hypertension was confirmed in a multivariate-adjusted model.

We previously examined genetic polymorphisms in two specific pathways as modifiers of the effects of air pollution within the NAS: oxidative stress and metal processing. We chose these pathways because prior work suggested *a*) that particulate air pollution generated oxidative stress, and *b*) that the soluble metal component of particulate air pollution was responsible for part of the effect. Specifically, we examined SNPs in genes related to oxidative stress as modifiers of the effects of particles on heart rate variability (Chahine et al. 2007), on endothelial markers (Madrigano et al. 2009), and on plasma homocysteine (Ren et al. 2010). We have also examined them as modifiers of the effects of ozone on lung function (Alexeeff et al. 2007), and we have examined associations with SNPs in metal-processing genes and heart rate variability (Park et al. 2006; Ren et al. 2010). Recently, we reported that polymorphisms in oxidative defense genes did not significantly modify the association of BC with BP, although there were trends in that direction (Mordukhovich et al. 2009). Taken together, these studies suggest that these pathways may be important for air pollution effects, although confirmation for other related outcomes and in other cohorts will be required.

In another recent paper, we demonstrated that BC exposure is associated with epigenetic changes—specifically, a reduction in LINE-1 methylation (Baccarelli et al. 2009). miRNAs are other important epigenetic mechanisms of gene expression control. Here we explored a novel pathway, polymorphisms in genes responsible for processing miRNAs, and found evidence for effect modification of BC on BP. Again, it will be important to determine whether these same polymorphisms modify effects on other end points and in other cohorts.

Although we believe our findings suggest altered susceptibility to BC exposures resulting from alterations in miRNA-processing related genotypes, we acknowledge that there are some limitations to our analysis. Despite these observations, we are not able to ascertain whether the polymorphisms described here modify the associations with BC because they are more susceptible to downregulation or because their basal activity makes individuals more susceptible to downstream changes in biological function. It is possible that both mechanisms may be at work. Additionally, it is also possible that observed associations could be attributable to LD between the genotyped SNPs and some other causal variant. For each of the SNP-by-BC interactions examined, we limited our investigation to associations under a recessive model of inheritance to maximize power to detect interactions, and therefore we may have overlooked some true associations that would only be observed under other additive or dominant models of inheritance. On the other hand, we also face the problem of multiple comparisons, as we examined multiple SNPs in the miRNA pathway. We tested interactions with 19 SNPs in this study, and multiple testing is subject to false positives. However, whereas studies of large numbers of genes incorporate corrections for multiple testing, we did not test separate independent hypotheses. Rather, we tested consistency of the pattern of association examined and not just the significance of the most significant association for the 10 genes examined.

We also acknowledge that there may be some misclassification of exposure in our analysis. Our study uses stationary measures of air pollution to represent personal exposures. Prior research indicates that when looking at longitudinal air pollution, most error is of the Berkson type. To the extent that this error is classical, simulation studies have shown that it is highly unlikely to bias away from the null even in the presence of covariates. This indicates that this exposure misclassification may lead to an underestimation of the health effects of air pollution (Zeger et al. 2000). In addition, several studies, including one conducted in the greater Boston area, have found that longitudinal measures of ambient particulate concentrations are representative of longitudinal variation in personal exposures (Rojas-Bracho et al. 2000). BC concentrations are spatially heterogeneous because of the numerous local (mobile) sources. Therefore, measurement error in our BC exposure metric would likely attenuate the true association. Given that we found significant positive associations for BC, it is unlikely that this error would affect our conclusions. Our study population is homogenous, consisting entirely of elderly men, and we have restricted our analysis to white individuals. The median distance of the participant homes from the central site monitoring station was 17.6 km. However, our findings are consistent with studies of particulate air pollution conducted in more heterogeneous populations.

## Conclusions

We have used a pathway approach to investigate how the processing of miRNAs may be a susceptibility factor for traffic particle–induced cardiovascular effects. Individual miRNAs may have multiple targets; more research is needed to address the relation between BC exposure and BP among diverse study populations and to clarify the mechanisms underlying the association between BC and BP. These results provide support for the hypothesis of the toxic effects of traffic pollution as measured by BC and contribute to growing understanding about the role of epigenetic modification and response to environmental stresses such as exposure to BC. In cardiovascular research, these results may be used to explore interactions with BC. Further investigations are needed to replicate our findings in other large cohort studies.

## Figures and Tables

**Table 1:** Descriptive statistics at baseline for participants included in analysis (*n* = 789).^a^

Study characteristic	Value
BP measurements	
SBP (mmHg)	132.0 ± 18.4
DBP (mmHg)	76.8 ± 10.9
Clinical measures	
Age at visit (years)	72.3 ± 7.5
BMI (kg/m^2^)	28.0 ± 4.1
Pulse (beats/min)	71.1 ± 8.1
Hypertension diagnosis	542 (81.8)
Hypertension medication use	478 (72.1)
Two or more drinks per day	199 (25.2)
Diabetes^b^	182 (23.1)
Education (years)	
≤ 12	280 (35.5)
13–16	372 (47.2)
> 16	137 (17.3)
Smoking status	
Never	237 (30.0)
Current	47 (6.0)
Former	505 (64.0)
Air pollution and weather data	
Seven-day moving average BC (μg/m^3^)	0.98 ± 0.415

Values are mean ± SD or no. (%).

a A total of 2,349 observations (one to six study visits) from 789 NAS participants were included in these analyses.

b Diabetes was classified as physician-diagnosed diabetes mellitus or fasting blood glucose > 126 mg/dL.

**Table 2:** Linear mixed-effects models estimating the change in BP associated with a 1-SD increase^a^ in BC.^b^

	Model 1^c^ mmHg (95% CI)	Model 2^d^ mmHg (95% CI)
SBP	3.52 (2.77–4.26)	3.04 (2.29–3.79)
DBP	2.72 (2.31–3.12)	2.28 (1.88–2.67)

a Corresponding to a 0.415-μg/m^3^ increase in 7-day average BC concentrations.

b A total of 2,349 observations from 789 NAS participants were included in these analyses.

c Model 1 was adjusted for age, BMI, smoking status, season, 7-day moving average of BC, and 7-day moving average of apparent temperature.

d Model 2 was adjusted for education, blood urea nitrate, age, BMI, pulse, smoking status, pack-years, median income, apparent temperature, alpha blocker use, beta blocker use, calcium channel blocker use, angiotensin receptor blocker use, ACE inhibitor use, diuretics, season, two or more drinks per day, and diabetes diagnosis.

**Table 3:** SNPs in miRNA processing genes included in analysis.

Gene^a^	RS number	SNP position	Alleles	Role	Amino acid change	MAF (%)
Gem-associated protein 3	rs197414	chr1:112110646	C/A	Coding exon	Arginine/serine	0.12
*(GEMIN3) (DDX20)*	rs197388	chr1:112099005	T/A	Promoter		0.18
	rs197412	chr1:112110476	T/C	Coding exon	Isoleucine/threonine	0.38
*AGO1 (EIF2C1)*	rs595961	chr1:36140367	A/G	Intron		0.15
*DROSHA (RNASEN)*	rs6877842	chr5:31568395	G/C	Promoter		0.18
Exportin 5 (*XPO5*)	rs11077	chr6:43598925	C/A	3′ UTR		0.40
*AGO2 (EIF2C2)*	rs4961280	chr8:141716596	C/A	Promoter		0.18
Tar-RNA binding protein 2 (*TARBP*)	rs784567^b^	chr12:52180732	C/T	Promoter		0.49
*DICER (DICER1)*	rs13078	chr14:94626500	T/A	3′ UTR		0.18
Gem-associated protein 4 (*GEMIN4*)	rs7813	chr17:594936	T/C	Coding exon	Cysteine/arginine	0.43
	rs1062923^c^	chr17:595817	T/C	Coding exon	Isoleucine/threonine	0.19
	rs3744741	chr17:595982	C/T	Coding exon	Arginine/glutamine	0.11
	rs4968104	chr17:596255	T/A	Coding exon	Valine/glutamic acid	0.27
	rs910925	chr17:596297	G/C	Coding exon	Glycine/alanine	0.42
	rs2740348	chr17:596685	G/C	Coding exon	Glutamic acid/glutamine	0.15
	rs910924	chr17:602670	C/T	Promoter		0.27
DiGeorge syndrome critical region gene 8 *(DGCR8)*	rs3757	chr22:18479331	G/A	3′ UTR		0.25
	rs1640299	chr22:18478359	G/T	3′ UTR		0.48
Ras-related nuclear protein *(RAN)*	rs14035	chr12:129927194	C/T	3′ UTR		0.28

Abbreviations: HWE, Hardy–Weinberg equilibrium; MAF, minor allele frequency; RS, reference SNP.

a Gene name (abbreviation), official gene symbol.

b HWE *p* = 0.08.

c HWE *p* = 0.05.

**Table 4:** Effect modification of the association between a 1-SD^a^ increase in BC concentrations and BP by gene variants related to miRNA processing under recessive models.^b^

			Sensitivity analyses		
		Results Δ mmHg (95% CI)	Model 2 *p*-interaction^c^	Model 1 *p*-interaction	IPW *p*-interaction^d^
DBP					
*DGCR8* rs1640299					
Wild-type and heterozygotes	590	2.57 (2.12 to 3.02)	0.006	0.002	0.004
Homozygous carriers	189	1.48 (0.78–2.18)			
*DICER* rs13078					
Wild-type and heterozygotes	738	2.32 (1.91 to 2.72)	0.017	0.03	0.008
Homozygous carriers	25	0.13 (−1.65 to 1.91)			
*GEMIN4* rs7813					
Wild-type and heterozygotes	638	2.44 (2.00 to 2.87)	0.02	0.04	0.006
Homozygous carriers	136	1.40 (0.60 to 2.21)			
*GEMIN4* rs910925					
Wild-type and heterozygotes	647	2.44 (2.02 to 2.87)	0.024	0.04	0.006
Homozygous carriers	134	1.43 (0.61 to 2.25)			

SBP					
*GEMIN3* rs197414					
Wild-type and heterozygotes	769	3.07 (2.32 to 3.82)	0.005	0.01	0.01
Homozygous carriers	10	−9.82 (−18.68 to −0.95)			
*GEMIN4* rs1062923					
Wild-type and heterozygotes	739	2.87 (2.10 to 3.65)	0.045	0.04	0.027
Homozygous carriers	37	5.58 (3.01 to 8.14)			

a A 1-SD increment in BC concentration is equal to 0.415 μg/m^3^.

b Adjusted for age (years), BMI, cigarette smoking status (never, current, former), pack-years smoked, blood urea nitrate, type 2 diabetes diagnosis, two or more alcoholic drinks per day, and current antihypertensive medication use (including angiotensin II receptor-, alpha-adrenoceptor, beta- or calcium channel blockers, diuretics, or ACE inhibitors) as well as apparent temperature, and season of clinical visit (spring: March–May; summer: June–August; autumn: September–November; winter: December–February).

c Two-sided *p*-value for interaction from SNP-by-BC coefficient.

d *p*-Value for interaction from SNP-by-BC coefficient for sensitivity analysis using IPW.
